# Child and adolescent psychiatry meets its challenges

**DOI:** 10.1007/s00787-022-02000-y

**Published:** 2022-05-13

**Authors:** Franziska Degenhardt

**Affiliations:** Department of Child and Adolescent Psychiatry, Psychosomatics and Psychotherapy, LVR Klinikum Essen, University of Duisburg-Essen, Rheinische Kliniken, Essen, Germany

Among adolescents (aged 10–24 years) living in EU Member States, mental disorders are compared to other non-communicable diseases such as chronic respiratory diseases or neurological disorders by far the leading cause of *years lived with disability* [1]. While the overall mortality rate decreased over the past 30 years, there is a rising trend of years of life lost due to mental disorders [1].

The ongoing Covid-19 pandemic and the brutal Russian invasion of Ukraine puts further stress on children and adolescents. The immediate and short-term impact of the ongoing Covid-19 pandemic on children are already well documented, while its longer-term consequences will eventually reveal themselves. In clinical practice, we have witnessed an unprecedented increase in hospital admissions due to eating disorders [2]. Furthermore, there is profound evidence that amid the Covid-19 pandemic, children and adolescents experience more stress, anxiety, and depression [3]. 

Child and adolescent psychiatry (CAP) must rise to these challenges and meet the mental health needs of this vulnerable population. Three years ago, representatives from associations relevant to CAP (i.e. Section on Child and Adolescent Psychiatry of the World Psychiatric Association, World Association for Infant Mental Health, Department of Mental Health and Substance Abuse of the World Health Organization, and others) met and discussed the future of CAP. Subsequently, four consensus priorities for our medical discipline for the next 10 years were published by Skokauskas et al. [4]. These included addressing the shortage of doctors trained in CAP and expanding research on the etiology of psychiatric disorders [4].

In contrast to the rising prevalence of mental disorders among adolescents [1], the number of child and adolescent psychiatrists is fairly low. According to the World Health Organization Mental Health Altas (2020), the median number of child and adolescent psychiatrists per 100 000 population (0–19 years old) ranges worldwide between 0.1 and 3.4 (European Region) [5]. CAP needs to work on a substantial expansion of the qualified workforce. Skokauskas et al. [4] suggested several strategies including: CAP specific training available to mental health professionals of various backgrounds (i.e. psychiatrists, nurses, social workers, psychologists, pediatricians); offering an appropriate salary; and greater access to training positions [4]. 

The latter is reported in this issue of *European Child and Adolescent Psychiatry.* Luisa Lázaro documented *the long road to the creation of the speciality of child and adolescent psychiatry in Spain* [6]. In 1950, The Spanish Association of Child and Adolescent Psychiatry was established. Despite tremendous efforts from various groups, including mental health professionals, Spanish child psychiatry associations, and citizen initiatives, it took more than 70 years to set up a specialty in CAP. Prior to this, a 4-month CAP training was included in the 4-year adult psychiatry curriculum of medical residency. Since 2008, individuals who wished to deepen their knowledge on CAP were able to expand CAP specific training to twelve months. Quite a few medical colleagues went abroad to further their training.

The new curriculum is estimated to be approved in 2023. Training in CAP will take 5 years, with educational aspects shared with adult psychiatry. It is estimated that the first (specifically) CAP trained doctors will complete their training in 2028. The author comments that this huge endeavour to offer excellent training for future CAP specialists in Spain might have been helped along by the mental health crisis created by Covid-19 [6].

In addition to enlarging the CAP workforce, Skokauskas and colleagues [4] identified *expanding research on the etiology of psychiatric disorders* as another CAP priority. It is well established, that psychiatric disorders are multifactorial with both genetic and non-genetic factors underlying their etiology. Their genetic architecture is complex with variants in likely thousands of genes contributing to the disorders pathophysiology. Genome-wide association studies (GWAS) focusing on common variants with small effect sizes provided unprecedented insight into the biological processes at play [7]. GWAS require enormous sample sizes to be adequately powered to detect variants of significance. For example, the most recent schizophrenia GWAS included more than 300,000 individuals. This tremendous effort resulted in the identification of common variant associations at 287 distinct genomic loci. Fundamental processes relevant to neuronal function were implicated [8]. The downside of these impressively large samples sizes is their lack of detailed phenotype information. Psychiatric disorders are clinically heterogeneous. Understanding not only their underlying etiology but also other clinically relevant factors (such as course of disorder, treatment response) will be important to improve our patients care.

These so far poorly understand aspects of psychiatric disorders are at the heart of the European Union funded Training Network CAPICE (*Childhood and Adolescence Psychopathology: unravelling the complex etiology by a large Interdisciplinary Collaboration in Europe)* [9]*.* In this issue of *European Child & Adolescent Psychiatry,* Rajula and colleagues describe a world-wide unique collection of eight population-based birth and childhood cohorts for which longitudinal, detailed phenotype data (environmental factors, lifestyle characteristics, emotional and behavioural symptoms) are available. In addition, for more than 70 000 individuals GWAS data and for more than 4 000 individuals epigenetic data are available. CAPICE aims to address the poorly understood interplay between non-genetic, environmental and genetic factors on the onset and course of disorder and the comorbidity patterns of childhood onset psychiatric disorders. The ultimate goal is to identify children at high risk for poorer outcomes and to improve future treatment and prevention options. CAPICE is an international training network for early career scientists. In addition to providing insight into the mechanisms underlying the phenotypic variance in affected children and adolescents, it builds up future leaders in CAP research. The cohorts included so far are mainly from Western Europe and Australia. To the authors knowledge no additional cohorts similar to those in CAPICE exist. However, if there are, they are welcome to join the Training Network [9].
